# Effect of Synthetic Fibers and Hydrated Lime in Porous Asphalt Mixture Using Multi-Criteria Decision-Making Techniques

**DOI:** 10.3390/ma13030675

**Published:** 2020-02-03

**Authors:** Carlos J. Slebi-Acevedo, Pedro Lastra-González, Miguel A. Calzada-Pérez, Daniel Castro-Fresno

**Affiliations:** 1 GITECO Research Group, Universidad de Cantabria, 39005 Santander, Spain; Carlosjose.slebi@unican.es (C.J.S.-A.); lastragp@unican.es (P.L.-G.); 2 GCS Research Group, Universidad de Cantabria, 39005 Santander, Spain; calzadam@unican.es

**Keywords:** PA mixture, fibers, hydrated lime, raveling, WASPAS, Taguchi

## Abstract

Porous asphalt is a type of mixture characterized by having high air void percentages that offers multiple benefits when used in wearing courses in terms of driving safety, water flow management, and noise reduction. However, the durability of porous asphalt (PA) mixtures is significantly shorter when compared to dense-graded asphalt mixtures. This study investigated the impact of polyolefin–aramid fibers and hydrated lime in the functional and mechanical performance of porous asphalt mixtures. A parametric study based on the concept of design of experiments was carried out through the Taguchi methodology. Accordingly, an experimental design was conducted based on the L18 full factorial orthogonal array. Three control factors—fiber content, binder content, and filler type—were included at various levels, and multiple responses including total air voids, interconnected air voids, particle loss in dry conditions, particle loss in wet conditions, and binder drainage were assessed experimentally. Signal-to-noise ratios were calculated to determine the optimal solution levels for each control factor for the multiple responses. In the second phase of the research, multi-criteria decision-making techniques—namely, criteria importance through inter-criteria correlation and weighted aggregated sum product assessment—were used to transform the multiple-response optimization problem into a single-unique optimization problem and to elaborate a preference ranking among all the mixture designs. The most significant levels for acquiring the optimum overall response value were found to be 0.05% for fiber content and 5.00% for binder content and mixed filler with hydrated lime.

## 1. Introduction

Porous asphalt (PA) mixtures in the last decades have become an attractive alternative to be implemented as wearing courses in the pavement structures due to the multiple benefits that they offer in terms of road safety and environmental aspects [1]. Due to the high porosity, these mixtures allow the flow of water through the mix to the sides of the road, minimizing the risk of aquaplaning, water splash, and spray effects [2]. Furthermore, PA mixtures positively help with driving safety since they reduce glare, improving night visibility as well as enhancing skid-resistance properties due to their macrotexture [3]. Other benefits include a reduction of traffic noise and a decrease of the urban heat island effect [4]. However, despite the advantages previously mentioned, the service life of the PA mixture is significantly shorter (i.e., 10–12 years) in comparison to dense-graded asphalt mixtures (i.e., 18 years) [5]. The high air void content of the mix means that the asphalt binder is more exposed to weather conditions; therefore, it is more likely to oxidize, causing a loss of adhesion in the aggregate–mortar matrix and loss of cohesion inside the asphalt mortar bridges, resulting in a reduction of raveling resistance [6,7,8,9].

In Spain and other European countries, the use of polymer-modified binder (PMB) is quite common in order to improve the durability of PA mixtures, due to the higher flexibility and elastic recovery that differentiates them from conventional bitumen [10,11,12]. However, the use of other additives such as fibers and hydrated lime could provide an alternative solution in order to increase the overall performance of PA mixtures. In dense-graded asphalt mixtures, promising results have been reported in the scientific literature with the use of fibers in terms of durability performance [13,14]. In general, it has been argued that fibers provide a three-dimensional reinforcement inside the mix, improving the tensile strength as well as fatigue properties and bringing ductility to the mixture [14,15,16,17]. Moreover, fibers act as a barrier inside the mix, preventing the formation and propagation of cracks [18]. In PA mixtures, it is well known that fibers are good stabilizers preventing binder leakage and so favoring the increase in binder content [19].

Although the majority of researches are focused on studying the improvement effects of one single type of fiber (i.e., steel [20], lignin [21], nylon [22], carbon [23], glass [24], basalt [25], jute [26]), few research efforts have been carried out incorporating hybrid fibers. Polyolefin–aramid (Pol-Aram) fiber is a set of two fibers that reinforce the mixture in different ways. On the one hand, polyolefin fiber works as a modifier of bitumen and dispersing agent [27,28], while aramid fibers, due to their high tensile strength and good thermal properties, contribute to support the tensile loads, forming a three-dimensional network inside the mix [28]. Good improvements have been reported in dense-graded asphalt mixtures using these hybrid fibers. For example, Kaloush et al. [29] studied the effect of fiber-reinforced asphalt concrete mixtures with Pol-Aram fibers using different characterization tests such as triaxial shear strength, crack propagation, flow number, and indirect tensile strength (ITS) tests. Based on the Mohr Coulomb failure envelope, the authors reported an increment in the cohesion of the mixture with added fibers without reducing the friction angle value. This result implies that fibers inside the mix provide an additional reinforcement while reducing the permanent deformation and increasing the shear strength. Moreover, higher modulus values and fatigue life at lower strain values were reported in the reinforced mixture when compared to the control mixture. Concerning ITS and fracture energy results, fiber-reinforced mixes exhibited increments of 25–50% for tensile strength and 50–75% for fracture energy as reported by the authors. Similar results were found by Klinsky et al. [28], who reported increments of about 20% for tensile strength in hot mix asphalt (HMA) with fibers in comparison to control mixtures. In addition, the authors reported better resistance to crack propagation with the addition of Pol-Aram fibers. In another study, Fazaeli et al. [30] evaluated the effect of Pol-Aram fibers in a warm mix asphalt (WMA) mixture. The authors reported a significant increment of 30% in rutting resistance at high temperatures when compared with a reference mixture. Similarly, the authors did not report problems of workability and compactability when Pol-Aram fibers were added. At lower material scale, the use of these mixed fibers were also investigated. Apostolidis et al. [31] performed pull-out and direct tension tests in order to evaluate the fiber–matrix interaction as well as the tensile strength properties in fiber-reinforced asphalt mortars. Based on the authors’ results, improvements in mechanical properties were identified with the addition of fibers.

Hydrated lime (HL) has proved to be of great interest as a potential filler for improving the durability of asphalt mixtures [32,33,34]. It has been documented that HL decreases the chemical aging of bitumen as well as increasing the resistance to frost and moisture damage [32,35]. Specifically, HL modifies the surface properties of the aggregate due to the free calcium ions and, therefore, improves the adhesion between asphalt binder and aggregates [32]. Furthermore, in clay materials, it modifies plastic properties, enhancing the moisture stability. Research literature has shown that HL improves particular properties in HMA such as the modulus, permanent deformation, fatigue life, and fracture toughness [36,37]. In fact, nowadays, the use of HL in HMA mixtures has become an accepted practice for many governmental road agencies with the intention of increasing the durability of the pavement [38].

While good mechanical characteristics have been proven in dense-graded asphalt mixtures with the addition of Pol-Aram fibers, there has been little research on PA mixtures with these fibers. In a previous research study, Slebi et al. [39] performed a study varying the binder and fiber content and taking into account two different types of binders (a conventional 50/70 penetration grade binder and a polymer-modified binder (PMB) 45/80–65). According to the experimental results, similar performance can be obtained using a conventional binder with Pol-Aram fibers or employing a polymer-modified binder. HL has long been identified in the scientific literature as a very beneficial admixture in bituminous mixtures. Furthermore, few studies have been reported that evaluate different additives simultaneously. In this study, the functional and mechanical performance of PA mixtures with Pol-Aram fibers and hydrated lime is investigated. The binder content (BC) and the fiber content (FC) as well as the filler type (FT) were selected as the three main factors in affecting the overall performance of modified PA mixtures. While the increment in binder and the inclusion of additives generally improve the durability of the mixture, the functional properties of the mixture such as porosity can be reduced. In the same way, since the fiber–binder proper quantities remain uncertain, the evaluation of one-factor-at-a-time is not the most appropriate methodology. The Taguchi design of experiments (DOE) is considered a robust technique that is capable of identifying the interactions presented in various control factors and recognizing the optimal levels for each control factor. The Taguchi approach was employed to set up the L18 orthogonal array and make the design of experiments easier and more consistent. Signal-to-noise ratios of the Taguchi method were used as objective functions to help in data analysis of individual responses such as total air voids, interconnected air voids, particle loss in dry conditions, particle loss in wet conditions, and binder drainage. Additionally, since more than one response is obtained, a multi-criteria decision-making analysis (MCDMA) was carried out in order to transform the multiple responses into a single optimal response and to elaborate a preference ranking among all the PA mixtures designed. More specifically, the criteria importance through inter-criteria correlation (CRITIC) technique was stablished for criteria elicitation, while the weighted aggregated sum product assessment (WASPAS) method was chosen for the preference ranking. The proposed novel technique deals with the efficiency of the assessment by applying CRITIC, since the presence of decision makers is not required to obtain the relative weights of different criteria and, so, facilitate automated decision making. Moreover, WASPAS is a robust, easily applicable decision-making tool that combines two MCDMAs, which are called the weighted sum model and weighted product model.

## 2. Materials and Experimental Plan

### 2.1. Materials

Conventional aggregates commonly used in Spain for the production of hot mix asphalt mixtures were used for the design of the PA mixture. Table 1 details the corresponding aggregate gradation adopted in the current study, fulfilling the requirements established in the Spanish standard [40]. Ophite (a type of igneous rock), with a density of 2.794 g/cm^3^ and Los Angeles abrasion value lower than 15% was used for the coarse fraction, while limestone with a density of 2.724 g/cm^3^ and sand equivalent of 78 was used as the fine fraction. In addition, two types of fillers were employed in this research. The first type comprises purely of limestone, whereas the other type of filler consists of a mix of limestone and hydrated lime (mixed filler). The proportion of HL contained in the PA mixture is 3.0% by weight of aggregates. The properties of hydrated lime according to the provider are shown in Table 2. The binder used in this study was a conventional 50/70 penetration graded binder. Its properties can be seen in Table 3.

**Figure 1:**
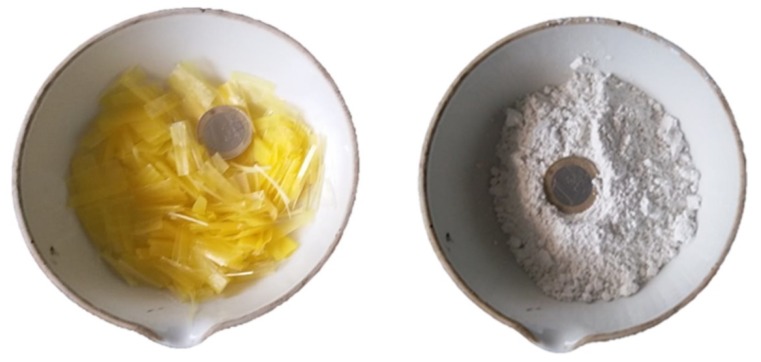
Modifiers used in this research. Pol-Aram fibers (**left**) and hydrated lime (**right**).

Concerning the type of fiber used in this research, a blend of synthetic fibers (polyolefin plus aramid) have been employed in the PA mixture due to the good results reported in the literature in dense-graded mixtures. The density of the set was determined according to the standard method UNE-EN 1097-6 given the value of 0.947g/cm^3^. The physical properties provided by the manufacturers are presented in Table 4. Moreover, an illustration of the additives is depicted in Figure 1.

### 2.2. Specimen Preparation and Experimental Plan

In this research, cylindrical specimens were compacted applying 50 blows per side following the European Standard method EN 12697–30. Fibers were added to the mix by the dry method. In other words, fibers were first added to aggregates and mixed until achieving a suitable homogeneous distribution of fiber–aggregate matrix; then, bituminous binder was added and blended so that the fiber–aggregate matrix was well coated by the bitumen. Concerning the experimental plan, different tests were performed from the functional and mechanical standpoint. In relation to functional properties, total air voids $T_{AV}$ were calculated by dimensional analysis according to EN 12697–8. In Spain, the $T_{AV}$ of the PA mixture is considered a parameter of some importance in order to measure the functional performance of the PA mixture. It is well known that a suitable content of air voids contributes to mitigating noise pollution and providing adequate permeability. However, in this research, the interconnected air voids $I_{AV}$ were also measured, following the procedure outlined by Montes et al. [41] and performed in other investigations [42]. As suggested by Alvarez et al. [7], interconnected air voids (also termed water-accessible voids) could be another possible indicator for the mix design and evaluation of the functional performance of the mixture. Concerning evaluation of the mechanical performance of the mixture, the Cantabro particle loss test in dry conditions was selected because it is one of the most common tests for measuring the durability of the mixture, and the raveling phenomenon was selected as well, which is one of the most common types of failure in PA mixture [43]. The particle loss test was performed at 25 °C following the European standards EN 12697–17. In this test, a compacted sample is placed in the Los Angeles Abrasion machine without steel spheres and subjected to 300 revolutions. Then, the percentage weight loss is then calculated by Equation (1).

(1) $$PL_{dry}\;\left(\%\right)=\frac{m_{i}-m_{f}}{m_{i}}\times 100$$

where $m_{i}$ is the initial weight; $m_{f}$ is the final weight of the mixture; and $PL_{dry}$ is the particle loss expressed in percentage.

In addition, since the PA mixtures are exposed to wet conditions, the particle loss test under wet conditions ($PL_{wet}$) was also conducted following the Spanish standard NLT 362/92. In this test, the samples were conditioned by immersion in water at 60 °C during 24 h and then placing them for another 24 h at 25 °C in dry conditions. Three replications were carried out for each test and for each PA mixture design, giving a total of 108 specimens. Finally, the binder drain down test was assessed in uncompacted PA mixture designs according to the European standard EN 12697–18 in order to analyze the potential effect of additives to be implemented as stabilizer agents.

## 3. Research Methodology

### 3.1. Parameters and Design of Experiments

Initially, the research started by selecting the additives that could impact positively the design of the porous asphalt mixture. Pol-Aram fibers and HL were selected as possible alternatives due to the good results reported in the literature. Since the objective of the present research is to maximize the functionality as well as durability, in this study, different responses were considered from the mechanical and functional point of view. Afterwards, a set of design parameters that affect the overall performance of the mixture was assigned. The parameters refer to the variables involved in the process that affect the different responses. Similarly, these parameters were grouped into three control factors: fiber content (FC), binder content (BC), and filler type (FT). Different numbers of levels were assigned to the control factors in the most significant and convenient manner. In this order of ideas, three levels were used for the first and second control factor and two levels were used for the third factor, as shown in Table 5. Once the parameters were established, the next step consisted in the elaboration of the design of experiments (DOE). In this research, the Taguchi methodology was selected because it is considered a suitable statistical technique for process optimization as well as for analyzing materials’ impact [44]. This methodology has been applied in various fields such as geopolymer concrete [45,46], polymer blended concrete [47], pervious Portland concrete pavement [48], self-compacting mortar [49], and now in PA mixtures reinforced with hybrid fibers and HL additives. Based on the concept of the orthogonal arrays that gives different combinations of the parameters, a full-factorial L18 (3 × 3 × 2) Taguchi orthogonal array was selected to conduct the experiments, as shown in Table 6.

Taguchi methodology allows the use of signal-to-noise (SN) ratios, which serve as objective functions in optimization that help in data analysis [48]. In other words, the purpose of employing SN ratios is to define which design parameters significantly affect the quality characteristic [50]. The highest value of SN ratio indicates the more positive impact of the design parameter on the response characteristics. Normally, there are three types of quality characteristics available in the analysis of SN ratio commonly known as *the smaller-the-better*, *the larger-the-better,* and *the-nominal-the-best* [51]. In this study, only *the smaller-the-better* and *the larger-the-better* quality characteristics were calculated depending on the response variable by using Equations (2) and (3), respectively.

(2) $$SN_{smaller-the-better}=\;-10\log\left(\frac{1}{n}\overset{n}{\underset{i=1}{\sum }}y_{i}^{2}\right)$$

(3) $$SN_{larger-the-better}=\;-10\log\left(\frac{1}{n}\overset{n}{\underset{i=1}{\sum }}\frac{1}{y_{i}^{2}}\right)$$

where $y_{i}$ corresponds to the observed response at the $i^{th}$ experiment and n corresponds to the number of observations of the experiment [51].

### 3.2. Multi-Criteria Decision-Making Analysis (MCDMA)

Multi-criteria decision-making (MCDM) methods help to identify the most promising alternatives contained in a set of alternatives based on previously established criteria [52]. Multiple MCDMA—Simple Additive Weighting (SAW), Weighted Product model (WPM), ELimination and Choice Expressing REality (ELECTRE), Gray Relational Analysis (GRA), Technique of Ordering Preferences by Similarity to Ideal solution (TOPSIS), Weighted Aggregated Sum Product Assessment (WASPAS), multi-criteria optimization and compromise solution (VIKOR), and Distance from Average Solution (EDAS)—have been applied in diverse fields such as material selection, military location, service quality, construction, and manufacturing processes [53,54,55,56,57,58,59]. However, limited literature has been found applying these techniques in combination with the DOE approach. When multiple responses are obtained, the implementation of (MCDM) methods are suitable in order to convert the multiple-response optimization problem into a single response optimization problem [60]. Among the methods most often applied in combination with the Taguchi DOE approach are the GRA and TOPSIS methods [50,61]. The combination of these robust techniques have been applied mainly in machining processing and manufacturing sector areas, mainly [60,62,63,64]. However, in road construction, few research studies have been done. In addition, little research effort has focused on applying other MCDMAs as was previously mentioned. Therefore, in this research, the Taguchi methodology is combined with the WASPAS method with the purpose of estimating the optimal parameters in one single decision-making process to establish appropriate responses for the PA mixture.

**Figure 2:**
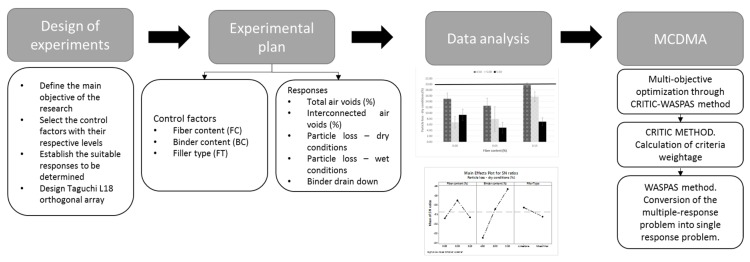
Structured framework of the current research.

To overcome the criteria elicitation drawback, decision makers have applied different approaches: the first ones include subjective weighting approaches that involve human participation for determining criteria weightage. The most commonly known are Analytic Hierarchy Process (AHP), Analytic Hierarchy Process under fuzzy environment (FAHP), and the best-worth method (BWM). Nonetheless, since these methods depend on human preferences, objective approaches are also attractive since the weights are established by mining the information contained in the original data [65]. The criteria importance through the inter-criteria correlation (CRITIC) method is referred to as an objective weighting approach that facilitates automated decision making [66]. This method was incorporated in this research due to the multiple datasets that are contained in it and because the weights derived from the approach enable appropriate management of the importance of each criterion selected and the conflict generated between criteria [66,67]. The following sections describe briefly the procedures of WASPAS and CRITIC approaches. The structured framework of this research is shown in Figure 2.

#### 3.2.1. WASPAS Method

This method introduced by Zavadskas et al. [68,69,70] in 2012 is considered one of the new powerful multi-criteria techniques because it combines two different multi-criteria methodologies commonly known as the Weighted Sum Model (WSM) and Weighted Product Model (WPM) [71]. This approach has been used in different decision-making fields such as the determination of manufacturing process conditions [72], industrial robot selection problems [73], optimal indoor environment selection [74], and construction site locations [75]. In this study, the authors employed this technique integrated with design of experiments to handle a parametric evaluation of different additives and quantities in different responses from a functional and mechanical point of view in a PA mixture. A brief description of the WASPAS method is presented in the following steps [73].

Step 1. Development of the decision/evaluation matrix showing the performance of different alternatives with respect to various criteria.

(4) $$X=\left[\begin{array}{cccc} x_{11} & x_{12} & \cdots & x_{1n}\\ x_{21} & x_{22} & \cdots & x_{2n}\\ \vdots & \vdots & \ddots & \vdots \\ x_{m1} & x_{m2} & \ldots & x_{mn} \end{array}\right]$$

where *m* corresponds to the number of alternatives and *n* is the number of criteria. $x_{ij}$ is the performance measured of $i^{th}$ alternative on $j^{th}$ criterion.

Step 2. Normalization of the decision matrix. In the WASPAS technique, the experimental data of the responses are first normalized in the range of zero to one. This procedure is required since the range and the units may differ between responses. In other words, it is necessary to transfer the original sequence of data in a comparable sequence of data [44]. Depending on whether data corresponds to beneficial or non-beneficial criteria, the following equations are employed.

For beneficial criteria:

(5) $${\bar{x}}_{ij}=\frac{x_{ij}}{\underset{i}{\max}x_{ij}}$$

For non-beneficial criteria:

(6) $${\bar{x}}_{ij}=\frac{\underset{i}{\min}x_{ij}}{x_{ij}}$$

where ${\bar{x}}_{ij}$ is a dimensionless number between 0 to 1 that corresponds to the normalized value of $x_{ij}$.

Step 3. Calculation of the first criterion of optimality (WSM) using the following equation:

(7) $$Q_{i}^{\left(1\right)}=\overset{n}{\underset{j=1}{\sum }}{\bar{x}}_{ij}w_{j}.$$

where $w_{j}$ is the weight of each criterion and $Q_{i}^{1}$ denotes the relative importance or significance of the $i^{th}$ alternative based on WSM. The alternative with the highest $Q_{i}^{\left(1\right)}$ score becomes the preferential choice according to WSM.

Step 4. Calculation of the second criterion of optimality (WPM) using the following expression:

(8) $$Q_{i}^{\left(2\right)}=\prod_{j=1}^{n}{\left({\bar{x}}_{ij}\right)}^{w_{j}}.$$

In this case, $Q_{i}^{\left(2\right)}$. denotes the relative importance or significance of the $i^{th}$ alternative based on WPM. Unlike the WSM approach, which strives toward additive aggregation properties, WPM is based on multiplicative aggregation properties. Additionally, the alternative with the highest $Q_{i}^{\left(2\right)}$ value is considered as the most suitable option.

Step 5. Application of a joint generalized criterion according to the WASPAS method. In order to unify the relative importance of WSM and WPM, a joint generalized criterion proposed by Zavadskas et al. [69,76] is applied as shown in the following equation:

(9) $$\begin{array}{c} Q_{i}=\lambda *Q_{i}^{\left(1\right)}+\left(1-\lambda \right)*Q_{i}^{\left(2\right)}=\lambda *\sum_{j=1}^{n}{\bar{x}}_{ij}w_{j}+\left(1-\lambda \right)*\prod_{j=1}^{n}{\left({\bar{x}}_{ij}\right)}^{w_{j}}\;\\ \lambda =0,0.1,0.2,\ldots ,1 \end{array}$$

where λ is a coefficient that linearly combines both methodologies. The preferred value is 0.5 since it gives equal relative importance to each methodology and, therefore, this value is employed in the present study. However, it is worth mentioning that through varying the λ value, it is possible to observe changes in the scores as well as in the ranking of alternatives. Consequently, when λ takes the value of 0, the alternatives are ranked according to the WPM, and when λ takes the value of 1, the preference ranking is selected according to the WSM.

#### 3.2.2. CRITIC Method

The CRITIC method was proposed by Diakoulaki et al. [77] in 1994. It was employed in this research since it aims at determining the objective weights of the different responses without considering human intervention. This method is based on the concept of contrasted intensity of each criterion and conflict assessment between criteria in the decision-making problem [66,78]. The procedure of this method is briefly described in the following stages as shown below. More details can be observed in [72,79].

Step 1. Construction of the initial decision/evaluation matrix involving the performance of the alternatives with respect to each criterion:

(10) $$X={\left[x_{ij}\right]}_{m*n}=\left[\begin{array}{cccc} x_{11} & x_{12} & \cdots & x_{1n}\\ x_{21} & x_{22} & \cdots & x_{2n}\\ \vdots & \vdots & \ddots & \vdots \\ x_{m1} & x_{m2} & \ldots & x_{mn} \end{array}\right]$$

where *m* is the number of alternatives and *n* the number of criteria. $x_{ij}$ represents the performance value obtained of the $i^{th}$ alternative in relation to the $j^{th}$ criterion.

Step 2. Normalization of the decision/evaluation matrix using the following equation:

(11) $${\bar{x}}_{ij}=\frac{x_{ij}-\underset{j}{\min}x_{ij}}{\underset{j}{\max}x_{ij}-\underset{j}{\min}x_{ij}}$$

where ${\bar{x}}_{ij}$ is the normalized performance value obtained for each alternative.

Step 3. Determination of the standard deviation of each criterion and the correlation coefficient among the set of criteria included in the decision/evaluation matrix. The weight on the $j^{th}$ criterion is determined as follows:

(12) $$w_{j}=\frac{c_{j}}{\sum_{j=1}^{n}c_{j}}$$

where $c_{j}$ is known as the amount of information contained in the $j^{th}$ criterion, which can be determined as follows:

(13) $$c_{j}=\sigma_{j}\overset{m}{\underset{j=1}{\sum }}\left(1-r_{ij}\right)$$

where $\sigma_{j}$ corresponds to the standard deviation of $j^{th}$ and $r_{ij}$ is the correlation coefficient relating two criteria. Based on the analysis of the procedure, it can be concluded that higher values of $c_{j}$ imply greater information obtained for each criterion, and therefore more relative significance of $j^{th}$ criterion is obtained [80].

## 4. Results and Discussion

### 4.1. Functional Performance

**Figure 3:**
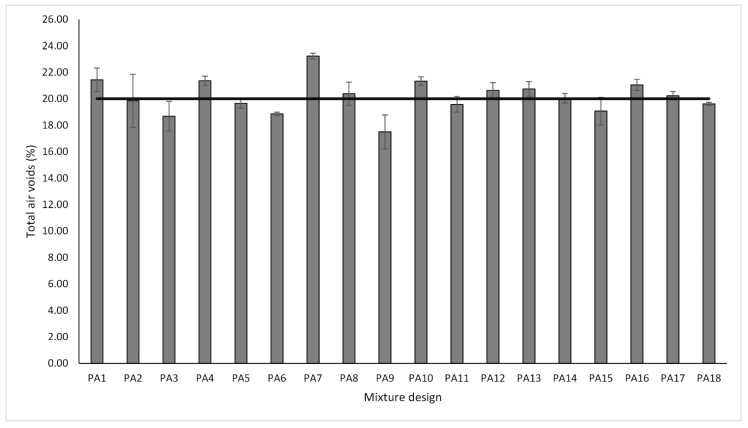
Total air voids response variable.

**Figure 4:**
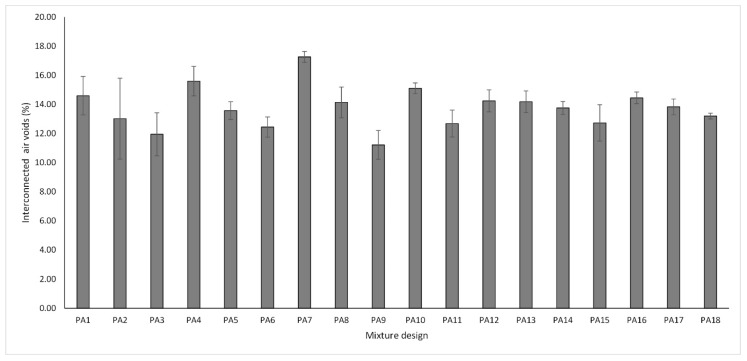
Interconnected air voids response variable.

**Figure 5:**
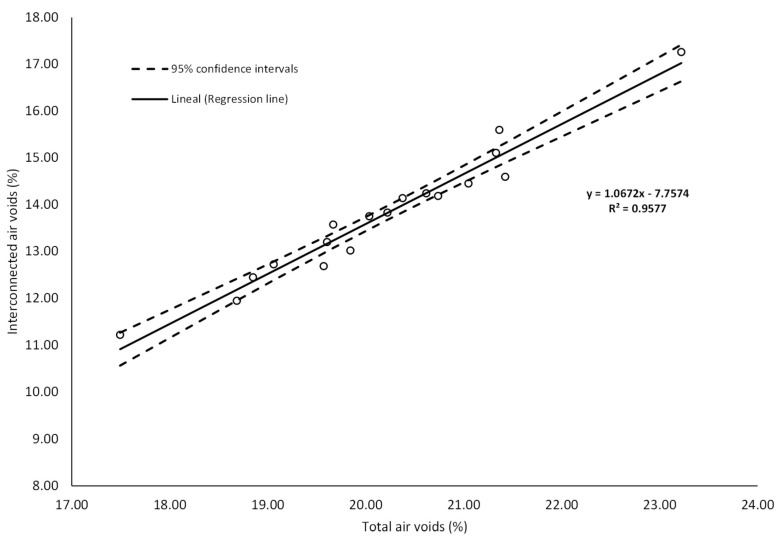
Linear relationship between total and interconnected air voids content.

It is well known that a high total number of air voids contributes to increasing the functionality of the PA mixture. However, the interconnected porosity among air voids also influences some properties of the mix, such as the hydraulic performance as well as noise mitigation properties. Therefore, in this research, $T_{AV}$ and $I_{AV}$ were selected as the main response variables to measure the functional performance of the PA mixture. The results of the total and interconnected air void characteristics are shown in Figure 3 and Figure 4, respectively. The error bars indicate the standard deviation of the mean of each PA mixture design. The range of values varied from 17.50% to 23.22% for $T_{AV}$ and from 11.22% to 17.26% for $I_{AV}$. Similarly, the mean values of all mixture designs for total and interconnected air voids were 20.18% and 13.77%, respectively. It is worth pointing out that there is a direct positive correlation between total and interconnected air voids with a Pearson coefficient of 0.98. Therefore, considering the relationship that exists between these two response variables, a linear regression model with a confidence interval of 95% was developed, as shown in Figure 5. Based on the data results, the model fits very well with a R-sq of 0.95 indicating a suitable prediction and, as a result, showing that higher values of total porosity imply higher values of interconnected porosity.

The open gradation curve was designed in order to provide a high total air voids content. Although, a minimum value of 20% of total air voids is required in order to guarantee proper hydraulic performance, skid resistance, and safety driving in Spain, other authors argued that PA mixtures with $T_{AV}$ greater than 18% are considered acceptable [7]. Overall, all the mixture designs fulfill the requirement of voids higher than 18% except for the PA9 mixture design that had an air void content of 17.50%. An explanation of the above could be that as the binder content increases, the binder film thickness increases, and therefore the air voids are reduced. Similarly, the fiber content contributes to retaining the bituminous binder and to keeping the binder film adhered to the aggregate.

**Figure 6:**
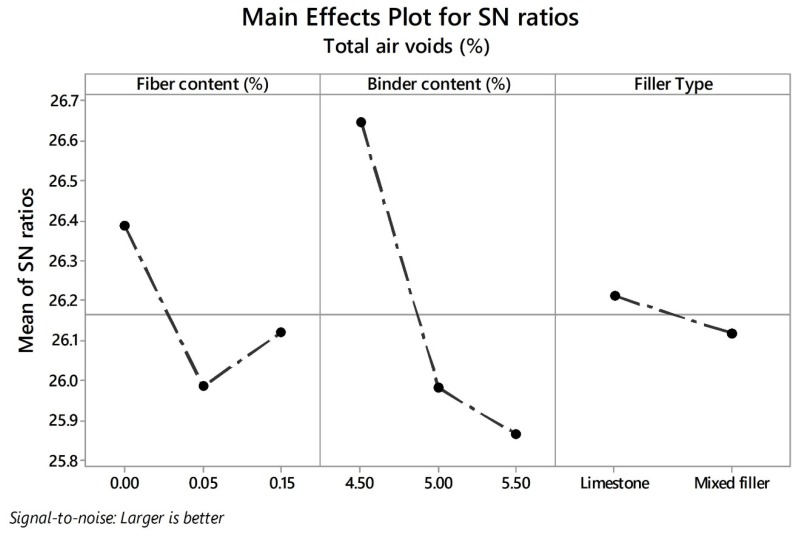
Effects of parameters on mean signal-to-noise (SN) ratios for $T_{AV}$.

**Figure 7:**
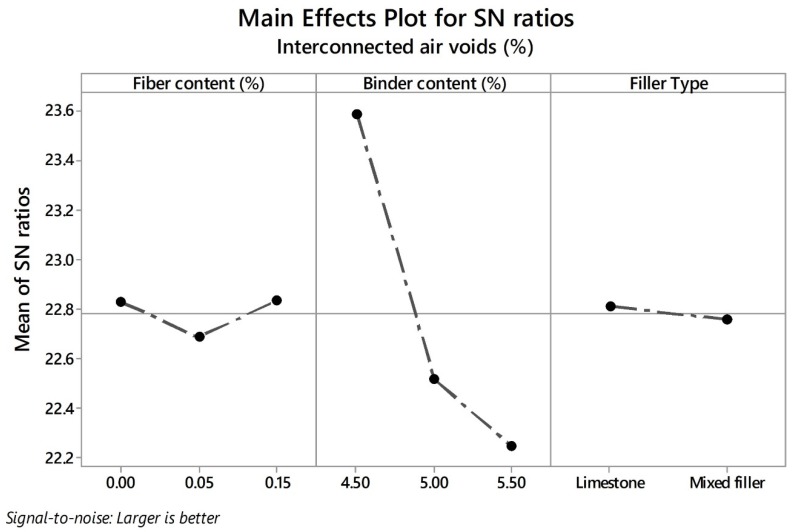
Effects of parameters on mean SN ratios for $I_{AV}$.

As higher values of $T_{AV}$ are relevant for functional quality improvement, the larger-the-better equation was used for the calculation of the SN ratios and analyzing the impact of the different control factors. In line with the Taguchi methodology, the best parameter for each control factor is the one that has the highest SN ratio value. Consequently, the parameters and SN ratios for the control factors giving the best $T_{AV}$ are shown in graph form in Figure 6. According to the results, the levels with their respective SN ratio for each control factor for the best $T_{AV}$ were identified for FC factor (level 1, SN ratio = 26.39), BC factor (level 1, SN ratio = 26.65), and for FT factor (level 1, SN ratio = 26.21). It means that the optimal solution values for total porosity can be obtained with a binder content of 4.50%, without fibers and pure limestone as a type of filler. Likewise, BC is the factor that most affects this property followed by fiber content, while the filler type is the least notable factor. With respect to interconnected air voids, there is no minimum target value in the norm; nonetheless, higher values of interconnected porosity imply better permeability rates. Therefore, the-larger-the-better equation was also used to calculate the SN ratios. Figure 7 depicts the SN ratios for $I_{AV}$. The parameters with their corresponding SN ratios per each control factor are as follows: for FC factor, level 3, SN ratio = 22.83; for BC factor, level 1, SN ratio = 23.59; and for FT, level 1, SN ratio = 22.81. This means that the optimal solution values for the interconnected porosity can be obtained with the same parameters as for the total air voids. An explanation of the above is the close relationship found between both variables. BC is the predominant factor concerning air voids characteristics; therefore, less binder content means a thinner binder film thickness and more pores inside the mix. Other researchers argued that the addition of fibers such as cellulose notably reduce the interconnected air voids [42]. However, due to the low quantity of fibers added, the interconnected porosity is not affected severely. In addition, since this type of fibers includes monofilaments, they possibly do not generate and obstruct the flow channels. It is noteworthy that the impact of fiber content on the interconnected air voids is less appreciable when compared to air voids response. Finally, FT was the factor with the least significance. Given that the hydrated lime has a specific gravity lower than limestone filler, a certain reduction in the air void characteristics of the mixture was expected. However, in this response, it was not distinguishable.

### 4.2. Mechanical Performance

**Figure 8:**
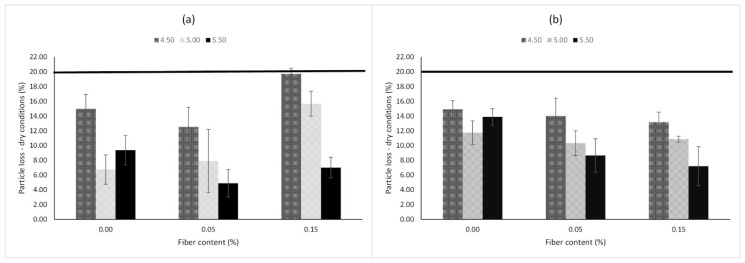
Particle loss in dry conditions of PA mixtures. (**a**) Limestone; (**b**) mixed filler.

Due to the large number of experiments that this research involved, a significant test had to be considered to assess the durability of the PA mixture. Since raveling is the most common type of failure present in this type of mixtures, the Cantabro particle loss test in dry conditions was performed. In addition, in order to measure water susceptibility, the Cantabro particle loss test was also carried out in wet conditions. Figure 8 plots the particle loss results in dry conditions $\left(PL_{dry}\right)$ for each type of filler used. Three replications for each mixture design were performed and the mean value was recorded. According to the literature and Spanish standards, a limit of 20% in the $PL_{dry}$ response is recommended [81]. In the current research, no mix design exceeded this value, and hence all mixture designs can be admissible. However, the results suggest that a suitable combination of additives has a significant influence on the raveling resistance. Concerning the limestone used as filler, improvements can be observed when adding fibers with a binder content of 5.50%. Specifically, the best $PL_{dry}$ response was obtained with 5.50% binder content and 0.05% Pol-Aram fibers. Based on the results, it can also be noted that an excess of fibers (i.e., 0.15%) is not suitable when the binder content is 4.50% or 5.50%. Fibers need to be properly coated in order to reinforce the PA mixture. With respect to the use of hydrated lime and limestone as mixed filler, improvements can be observed by adding fibers with either 5.00% or 5.50% of binder content. Although the best raveling resistance in terms of particle loss for this group was obtained adding 0.15% Pol-Aram fibers with a binder content of 5.50%, better results were obtained with limestone filler, with a binder content of 5.50% and fiber content of 0.05%.

**Figure 9:**
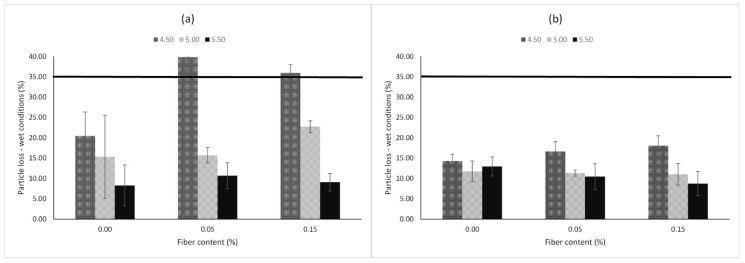
Particle loss in wet conditions of PA mixtures. (**a**) Limestone. (**b**) Mixed filler.

**Figure 10:**
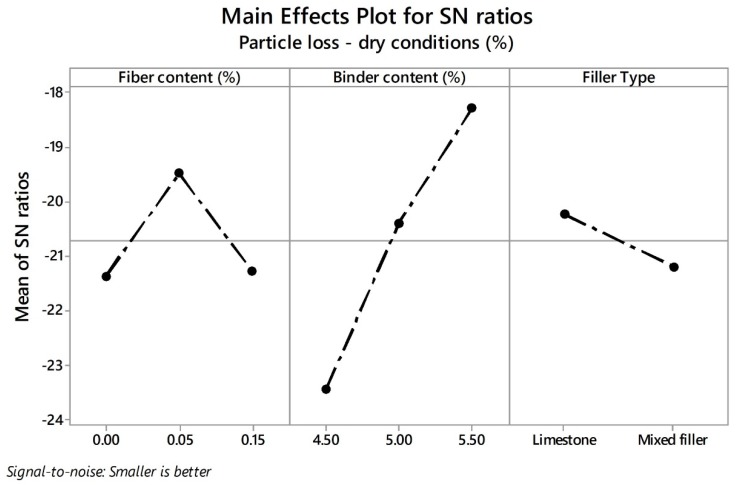
Effects of parameters on mean SN ratios for $PL_{dry}$.

**Figure 11:**
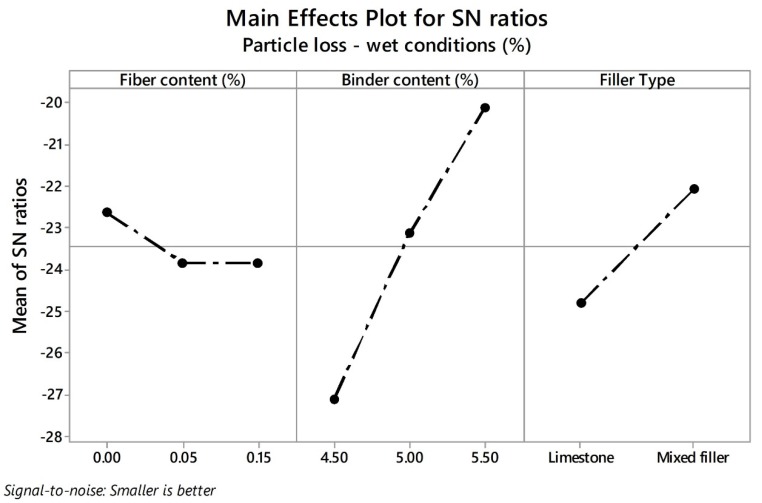
Effects of parameters on mean SN ratios for $PL_{wet}$.

In relation with the particle loss in wet conditions $\left(PL_{wet}\right)$ response variable, Figure 9 shows the mean values of the PA mixture designs for each type of filler. Based on the Spanish standard, a maximum value of 35% is allowed as the $PL_{wet}$ response in order to guarantee proper durability. Concerning mixtures prepared with limestone as filler, the addition of fibers with low quantities of bitumen is not recommended (i.e., 4.50%). Similarly, there are not notable improvements adding fibers with greater quantities of bitumen. On the other hand, PA mixtures with mixed filler exhibited good raveling resistance under the action of water compared to mixtures with limestone as pure filler. In addition, there are no notable improvements in the $PL_{wet}$ response from adding fibers. In order to investigate the influence of control factors with their respective parameters on the $PL_{dry}$ and $PL_{wet}$ response variables, the SN ratios were plotted as can be observed in Figure 10 and Figure 11, respectively. The lowest values of particle loss in dry and wet conditions are desirable in order to obtain the best durability of the mix. Therefore, the-smaller-the-better equation was used for calculation.

Similarly to the air void characteristics case, the highest SN ratio values of each parameter of each control factor represent the optimal solution value of each response. Accordingly, the levels and the corresponding SN ratios for the control factors with the optimal solution values for $PL_{dry}$ were identified as the FC factor (level 2, SN ratio = −19.49), BC factor (level 3, SN ratio = −18.30), and FT factor (level 1, SN ratio = −20.23), which means that the optimum values can be obtained with a fiber content of 0.05%, a binder content of 5.50%, and limestone as filler. The control factor with the highest incidence corresponds to the binder content followed by the fiber content and filler type. The higher the binder content, the greater thickness of the binder film formed around the aggregate, which leads to an increment in the adhesive forces in the binder–aggregate interface. The addition of fibers also contributes to reinforcing the mixture. Previous research argued that fibers can support the tensile strengths generated in the mixture [22]. Additionally, it could be said that fibers also help to reinforce the PA mixture, strengthening the cohesive forces inside the mortar matrix. Apostolidis et al. [31] studied the effect of synthetic fibers at the asphalt mortar scale. The authors reported improvements in the mechanical characteristics of the mortar when fibers were added. Finally, the type of filler is the least significant parameter with respect to $PL_{dry}$ response. In other words, the raveling resistance in dry conditions will not vary notably depending on the filler type employed.

Similarly to the $PL_{dry}$ response, the levels with their corresponding SN ratios for $PL_{wet}$ response variable were calculated as follows: for FC factor (level 1, SN ratio = −22.63), for BC factor (level 3, SN ratio = −20.09), and for FT factor (level 2, SN ratio = −22.07). In other words, the optimal solution values for particle loss in wet conditions can be obtained using a mixed filler and binder content of 5.50%. In this response, BC is the most significant factor followed by the type of filler and the fiber content. Due to the high air void content, the bituminous binder is quite prone to oxidation by the action of water. A greater amount of binder implies a greater increase in the thickness of the binder film. However, an excess of binder content causes a risk of binder drain down, which will be discussed in the next section. As suggested by Jaya and Asif [82], thicker asphalt binder films produce more flexible and durable mixes, whereas thin films produce more brittle mixes that are more prone to ravel. On the other hand, the use of HL as part of the filler showed an improvement in raveling resistance by moisture damage. HL is composed of calcium hydroxide, which promotes the precipitation of calcium ions over the surface of the aggregate, and so favors the bonding between aggregate and bituminous binder [83]. Moreover, the high values of dry porosity of HL when compared to other mineral fillers could positively influence the raveling resistance. As suggested by Lesueur et al. [32], due to the high dry porosity, the internal pores of the HL particles can be filled with bitumen and possibly contribute to better interlocking with the mortar matrix. Moreover, this “active” filler, which is confirmed in the literature, has the potential to reduce the oxidation as well as the aging in the bitumen [84]. Finally, although the fiber content was the control factor with the lowest impact, slight improvements were observed with high values of binder content (i.e., 5.50%). This suggests that fibers must be properly coated by the bitumen so that they can bond firmly with the aggregate, preventing particle loss.

### 4.3. Binder Drain Down

**Figure 12:**
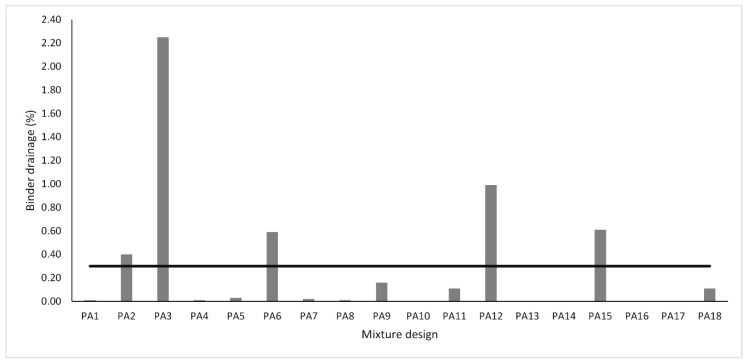
Binder drainage results according to the mesh basket drain down test.

**Figure 13:**
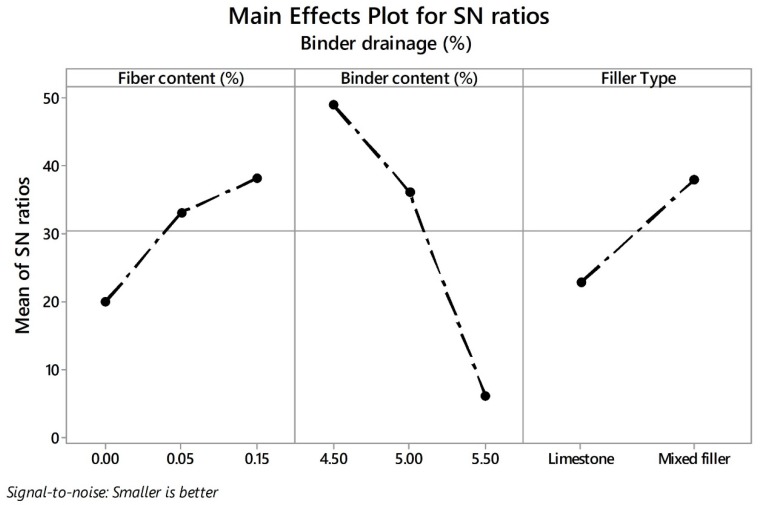
Effects of parameters on mean SN ratios for binder drainage (BD).

This test provides an assessment of the binder drainage potential of a PA mixture during the mixing process, transport, or field placement. The results for all PA mixture designs were plotted in Figure 12. According to the literature, a limit value of 0.3% is recommended for PA mixtures [85,86]. As expected, the mixtures that presented the highest binder drainage correspond to those with the highest binder content. On the other hand, mixtures with a binder content of 4.5% did not register any drain down drawback. Concerning fibers, this additive has a stabilizing effect since mixtures modified with fibers presented lower binder drainage values. Considering PA mixtures without fibers, it can be noted that mixtures with a mixed filler presented a drain down reduction when compared with those mixtures that only used limestone as filler. This result suggests that the addition of HL could contribute to retaining bitumen and thus increase the binder drainage. For binder drainage response, lower values are suitable. Therefore, a smaller-the-better equation was employed to calculate the SN ratios. Figure 13 shows in graph form the SN ratios of BD response. In agreement with this, the levels and SN ratios that give the lowest BD were identified for FC factor (level 3, SN ratio = 38.18), for BC factor (level 1, SN ratio = 48.99), and for FT factor (level 2, SN ratio = 38.01). According to the results, the control factors that most influence this response correspond to BC followed by FC, and FT had the least influence. Although cellulose fiber is the most common type of fiber to prevent the binder leakage due to the surface area, Pol-Aram fibers have worked properly and also perform very well as a stabilizer agent. In the same way, the internal porosity of HL is much higher than that of conventional fillers. Probably, these internal voids contribute to absorbing more binder and, so, to preventing the binder leakage.

### 4.4. CRITIC–WASPAS Methodology

The optimal design of a PA mixture implies the optimization of different responses. In other words, a PA mixture must fulfill different requirements from functional and mechanical standpoints. In the same way, when several parameters are involved in many criteria, determining the most suitable alternative could be considered a complex task. Due to the above, the CRITIC–WASPAS approach was integrated with the Taguchi methodology in order to turn the multiple-response optimization problem into a single response optimization problem. Firstly, the weights of different responses must be assigned. Although it is quite common to designate equal weights for all responses [62], using an appropriate weighting criterion is more beneficial in obtaining a good solution. The CRITIC method was considered a reasonable technique to take advantages of the data information according to the contrasted intensity of the response and conflict assessment between criteria, as explained in Section 3.2.2. First, the decision matrix is normalized by using Equation (11), as can be observed in Table 7. The standard deviation (SD) values for all criteria are shown in the last row of Table 7. Then, the Pearson correlation coefficient between criteria is calculated, as shown in Table 8. Afterwards, the criteria weighting were determined by using Equations (13) and (14). Table 9 shows the final weights for the different responses. According to the CRITIC method, particle loss in dry and wet conditions was the response with the highest weights whose values correspond to 0.24 and 0.26, respectively. Subsequently, air void characteristics and binder drainage response obtained similar criteria weighting with an approximate value of 0.17.

**Figure 14:**
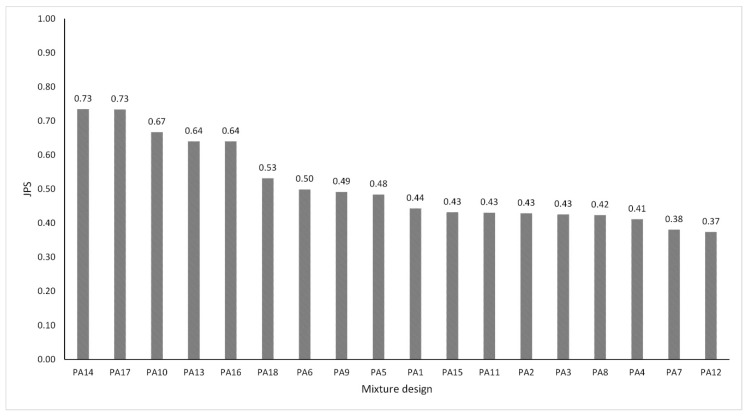
Joint Performance Score (JPS) values and preference ranking according to the WASPAS method.

The following step is to find the preference ranking of all PA mixture designs according to the WASPAS technique. A Joint Performance Score (JPS) value for each alternative is determined from the response variables employing the WASPAS method with the help of Equation (4) through Equation (9). First, the normalized decision matrix for beneficial and non-beneficial criteria is obtained by using Equations (5) and (6), as shown in Table 10. Subsequently, by using Equations (7) and (8), the weighted normalized sum model and weighted normalized product model were calculated as can be observed in Table 11 and Table 12, respectively. Finally, the JPS values of all PA mixture designs were calculated by using Equation (9) in order to do the preference ranking, as shown in Figure 14. The PA mixture alternative with the highest JPS value is considered to be the optimal parameter combination among all the experiments carried out according to the WASPAS method. In agreement with the results, PA 14 and PA 17 top the ranking, since they have the best multiple objective responses among the 18 experiments considered. In this way, a PA mixture with mixed filler, a binder content of 5.50%, and adding 0.05% of Pol-Aram fibers exhibited the best overall performance. In contrast, the PA12 mixture design obtained the lowest JPS value.

**Figure 15:**
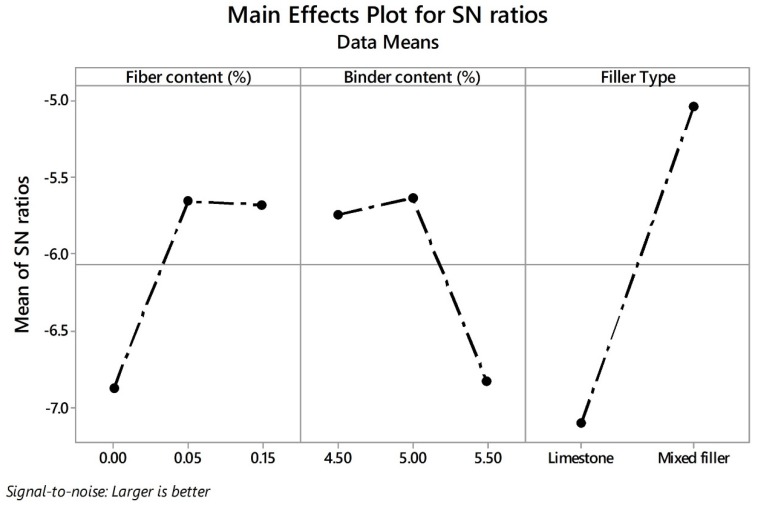
Effects of parameters on mean SN ratios for JPS value.

Similarly to individual responses, the SN ratios based on the Taguchi concept were calculated in order to identify the optimal input parametric values. In this sense, the-larger-the-better equation was used to calculate the SN ratios that are displayed in graph form in Figure 15. According to the results, the control factors that most influenced the overall response were the filler type, followed by the fiber content and the binder content. The addition of hydrated lime notably influenced the $PL_{wet}$ response as well as the BD response and did not cause a significant negative impact on air voids characteristics. Actually, the tendency is toward the use of hydrated lime in hot mix asphalt [87]. In European countries, the use of HL in asphalt mixtures is increasing, and in some countries such as the Netherlands, the use of HL is specified in the design of PA mixtures [87]. The addition of fiber notably influenced the $PL_{dry}$ and binder drainage responses. Although slightly reducing the $T_{AV}$ response, it did not register a negative impact on $I_{AV}$ response. Finally, the binder content factor positively influenced the mechanical performance of the mixture. However, it generates reductions in the air void characteristics and negatively affects the binder drainage response. Moreover, the optimal input parameters were identified on the second level for the three control factors. The above means that the most significant levels for acquiring the optimal overall response value were recognized as 0.05% for FC, 5.00% for BC, and mixed filler for FT.

**Figure 16:**
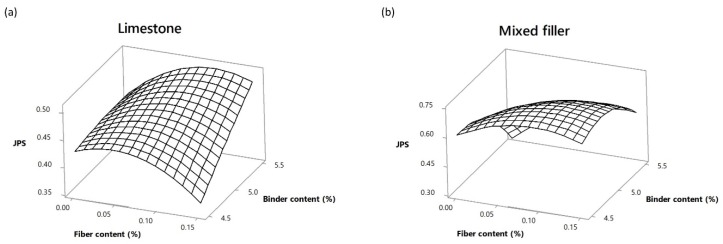
Fiber–binder interaction effect on JPS value. (**a**) Limestone. (**b**) Mixed filler.

Additionally, the fiber–binder interaction effect on the JPS value was plotted depending on the type of filler employed, as shown in Figure 16. For the case of limestone as filler, low binder quantities with higher fiber contents decrease the JPS value, whereas high quantities of binder content and the addition of fibers leads to an increment in the JPS value. The maximum JPS value was reached adding 0.05% of Pol-Aram fibers and a binder content of 5.00%. On the other hand, for mixed filler, the excess of binder without fibers decreases the overall performance. In contrast, low binder content without fibers had a positive effect on the overall performance.

Taking into account that JPS values can be considered a suitable indicator of the overall performance of PA mixtures, a linear plus interaction regression model was applied to integrate the three control input factors. This means that the independent variables were fiber content (FC), binder content (BC), and filler type (FT), whereas the dependent variable was the JPS value. This model was chosen due to its simplicity and because it can be applied easily. When different input parameters are involved in a decision-making process, simple and significant mathematical models are preferred. The predictive equation for the linear plus interaction regression model is given as follows:

(14) $$\begin{array}{c} JPS=0.472-4.960\times FC\left(\%\right)-0.0052\times BC\left(\%\right)+1.389\times FT+\\ 0.983\times FC\left(\%\right)\times BC\left(\%\right)+0.922\times FC\left(\%\right)\times FT-0.2635\times BC\left(\%\right)\times FT. \end{array}$$

**Figure 17:**
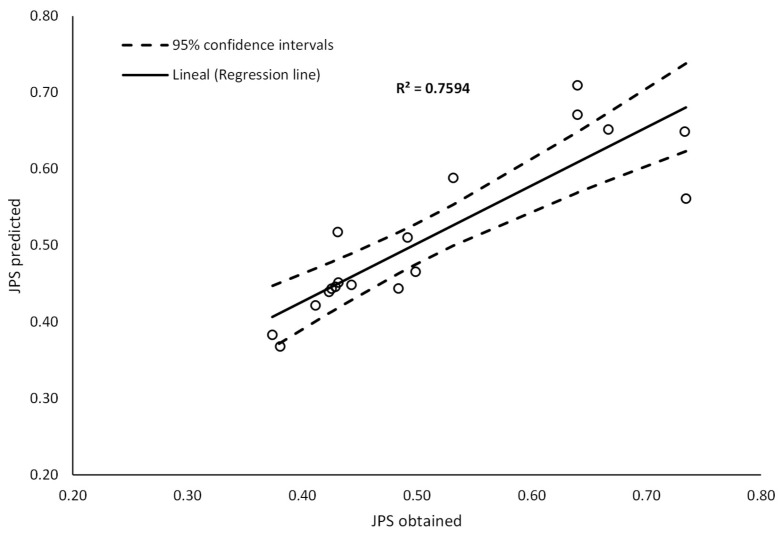
Comparison between JPS values obtained from multi-criteria decision-making analysis (MCDMA) and regression model.

The comparison between JPS values obtained from MCDMA and those predicted by the regression model is shown in Figure 17. To validate the model, analysis of variance with a confidence interval of 95% was carried out as shown in Table 13. The *p*-values < 0.05 suggest the significance of the model as well as its linear and interaction components. In addition, the $R^{2}$ value for JPS was found to be 75.94%. Good relation was also found between predicted values and values obtained from MCDMA with a mean error of 7.15%.

## 5. Conclusions

This study investigated the impact of Pol-Aram fibers and hydrated lime on the functional and mechanical performance of PA mixtures. A parametric study based on the concept of design of experiments was carried out through the Taguchi methodology. Accordingly, an experimental design was developed based on the L18 full factorial orthogonal array. Three control factors—that is, fiber content, binder content, and filler type—were included at various levels and multiple responses including total air voids, interconnected air voids, particle loss in dry conditions, particle loss in wet conditions, and binder drainage were assessed experimentally. Signal-to-noise ratios were calculated to determine the optimal solution levels for each control factor for the multiple responses. In the second phase of the research, multi-criteria decision making techniques—that is, criteria importance through inter-criteria correlation and weighted aggregated sum product assessment—were used to transform the multiple-response optimization problem into a single optimization problem and to elaborate a preference ranking among all the mixture designs. The conclusions obtained from the results and the analysis of experiments are given below:

- Concerning functional performance, total and interconnected air voids response were calculated for all PA mixture designs. According to the results, the binder content is the most influential factor in both responses. Adding fibers slightly reduces the total air voids and does not notably affect the interconnected air voids characteristics. Moreover, the type of filler is considered the least influential factor.
- As for mechanical performance, in this study, particle loss in dry and wet conditions were the main responses considered. For $PL_{dry}$ response, adding 0.05% of Pol-Aram fibers with 5.50% of binder and limestone as type of filler is the optimal parameter solution. For $PL_{wet}$ response, 5.50% of binder content and mixed filler with hydrated lime improve this response, while the least influence is shown by the addition of fibers.
- For binder drainage response, the set of polyolefin–aramid fibers as well as hydrated lime contributed to preventing binder leakage in the mix.
- In relation to criteria elicitation, the CRITIC objective weighting approach was contemplated. Consequently, the mechanical performance criteria were assigned with a weighting of approximately 50%, while air void characteristics and binder drainage responses were assigned the remaining 50%.
- According to the JPS values attained from the WASPAS approach, the PA14 mixture design was ranked as the best alternative. Adding 0.05% of Pol-Aram fibers with a binder content of 5.00% and mixed filler with HL performs very well in all the multiple responses. Similarly, according to the SN ratios for the JPS values, the most significant levels for acquiring the optimal overall response value were found to be 0.05% for FC, 5.00% for BC, and mixed filler for FT, coinciding with the parameters obtained from the PA14 mixture.

## Figures and Tables

**Table 1:** Gradation of aggregates used in this research.

Sieve Size (mm)	Upper Limit (%)	Gradation Used (%)	Lower Limit (%)
22	100	100	100
16	100.0	95.0	90.0
8	60.0	50.0	40.0
4	27.0	20.0	13.0
2	17.0	13.5	10.0
0.5	12.0	8.5	5.0
0.063	6.0	4.5	3.0

**Table 2:** Physical and chemical properties of hydrated lime (HL).

Properties	Value
Density (g/cm^3^)	1.959
CaO content (%)	≥90
MgO content (%)	≤5
CO_2_ content (%)	≤4
Remaining in sieve 0.2 mm (%)	≤2
Remaining in sieve 0.09 mm (%)	≤7

**Table 3:** Main binder properties.

Test	Standard Method	Value
Penetration at 25 °C (mm/10)	EN 1426	57.00
Specific gravity	EN 15326	1.035
Softening point (°C)	EN 1427	51.60
Fraass brittle point (°C)	EN 12593	−13.00

**Table 4:** Characteristics and properties of polyolefin–aramid (Pol-Aram) fibers.

Fiber	Aramid	Polyolefin
Form	Monofilament	Serrated
Color	Yellow	Yellow
Density (g/cm^3^)	1.44	0.91
Length (mm)	19	19
Tensile strength (MPa)	2758	483
Decomposition temperature (°C)	>450	157
Acid/Alkali resistance	Inert	Inert

**Table 5:** Control factors with their corresponding levels.

Factors	Level 1	Level 2	Level 3
FC: Fiber content (%)	0.00	0.05	0.15
BC: Binder content (%)	4.50	5.00	5.50
FT: Filler type	Limestone	Mixed filler	-

**Table 6:** Full factorial design with Taguchi orthogonal array L18.

Mixture Design	Fiber Content (%)	Binder Content (%)	Filler Type
PA1	0.00	4.50	Limestone
PA2	0.00	5.00	Limestone
PA3	0.00	5.50	Limestone
PA4	0.05	4.50	Limestone
PA5	0.05	5.00	Limestone
PA6	0.05	5.50	Limestone
PA7	0.15	4.50	Limestone
PA8	0.15	5.00	Limestone
PA9	0.15	5.50	Limestone
PA10	0.00	4.50	Mixed filler
PA11	0.00	5.00	Mixed filler
PA12	0.00	5.50	Mixed filler
PA13	0.05	4.50	Mixed filler
PA14	0.05	5.00	Mixed filler
PA15	0.05	5.50	Mixed filler
PA16	0.15	4.50	Mixed filler
PA17	0.15	5.00	Mixed filler
PA18	0.15	5.50	Mixed filler

**Table 7:** Normalized decision matrix according to criteria importance through inter-criteria correlation (CRITIC) methodology.

Design	T_AV_ (%)	I_AV_ (%)	PL_DRY_ (%)	PL_WET_ (%)	BD (%)
PA1	0.69	0.56	0.28	0.61	1.00
PA2	0.41	0.30	0.87	0.78	0.82
PA3	0.21	0.12	0.70	1.00	0.00
PA4	0.68	0.72	0.49	0.00	1.00
PA5	0.38	0.39	0.80	0.76	0.99
PA6	0.24	0.20	1.00	0.92	0.74
PA7	1.00	1.00	0.00	0.12	0.99
PA8	0.50	0.48	0.27	0.54	1.00
PA9	0.00	0.00	0.86	0.97	0.93
PA10	0.67	0.64	0.32	0.81	1.00
PA11	0.36	0.24	0.54	0.89	0.95
PA12	0.55	0.50	0.39	0.85	0.56
PA13	0.57	0.49	0.39	0.74	1.00
PA14	0.44	0.42	0.63	0.90	1.00
PA15	0.27	0.25	0.75	0.93	0.73
PA16	0.62	0.54	0.44	0.69	1.00
PA17	0.48	0.43	0.60	0.91	1.00
PA18	0.37	0.33	0.84	0.99	0.95
SD	0.23	0.23	0.26	0.28	0.25

**Table 8:** Correlation coefficient values between responses.

	T_AV_ (%)	I_AV_ (%)	PL_DRY_ (%)	PL_WET_ (%)	BD (%)
**T_AV_ (%)**	1.00	0.98	−0.85	−0.75	0.40
**I_AV_ (%)**	0.98	1.00	−0.82	−0.81	0.44
**PL_DRY_ (%)**	−0.85	−0.81	1.00	0.63	−0.27
**PL_WET_ (%)**	−0.75	−0.81	0.63	1.00	−0.36
**BD (%)**	0.40	0.44	−0.27	−0.36	1.00

**Table 9:** Weights assignment for different responses.

Criteria	T_AV_ (%)	I_AV_ (%)	PL_DRY_ (%)	PL_WET_ (%)	BD (%)
**Cj**	0.95	0.98	1.40	1.48	0.95
**Wj**	0.16	0.17	0.24	0.26	0.16

**Table 10:** Normalized decision matrix according to weighted aggregated sum product assessment (WASPAS) methodology.

Design	T_AV_ (%)	I_AV_ (%)	PL_DRY_ (%)	PL_WET_ (%)	BD (%)
PA1	0.92	0.85	0.31	0.40	0.10
PA2	0.85	0.75	0.72	0.54	0.00
PA3	0.80	0.69	0.52	1.00	0.00
PA4	0.92	0.90	0.39	0.21	0.10
PA5	0.85	0.79	0.62	0.53	0.03
PA6	0.81	0.72	1.00	0.77	0.00
PA7	1.00	1.00	0.25	0.23	0.05
PA8	0.88	0.82	0.31	0.36	0.10
PA9	0.75	0.65	0.70	0.91	0.01
PA10	0.92	0.88	0.33	0.58	1.00
PA11	0.84	0.73	0.42	0.70	0.01
PA12	0.89	0.83	0.35	0.64	0.00
PA13	0.89	0.82	0.35	0.50	1.00
PA14	0.86	0.80	0.47	0.73	1.00
PA15	0.82	0.74	0.56	0.79	0.00
PA16	0.91	0.84	0.37	0.46	1.00
PA17	0.87	0.80	0.45	0.75	1.00
PA18	0.84	0.76	0.68	0.95	0.01

**Table 11:** Weighted normalized sum model.

Design	T_AV_ (%)	I_AV_ (%)	PL_DRY_ (%)	PL_WET_ (%)	BD (%)	Performance Score
PA1	0.15	0.14	0.08	0.10	0.02	0.49
PA2	0.14	0.13	0.18	0.14	0.00	0.58
PA3	0.13	0.12	0.13	0.26	0.00	0.63
PA4	0.15	0.15	0.09	0.05	0.02	0.47
PA5	0.14	0.13	0.15	0.14	0.01	0.57
PA6	0.13	0.12	0.24	0.20	0.00	0.70
PA7	0.16	0.17	0.06	0.06	0.01	0.46
PA8	0.14	0.14	0.08	0.09	0.02	0.47
PA9	0.12	0.11	0.17	0.23	0.00	0.64
PA10	0.15	0.15	0.08	0.15	0.16	0.69
PA11	0.14	0.13	0.10	0.18	0.00	0.55
PA12	0.15	0.14	0.09	0.16	0.00	0.54
PA13	0.15	0.14	0.08	0.13	0.16	0.67
PA14	0.14	0.14	0.12	0.19	0.16	0.75
PA15	0.14	0.13	0.14	0.20	0.00	0.60
PA16	0.15	0.14	0.09	0.12	0.16	0.67
PA17	0.14	0.14	0.11	0.19	0.16	0.75
PA18	0.14	0.13	0.16	0.24	0.00	0.68

**Table 12:** Weighted normalized product model.

Design	TAV (%)	IAV (%)	PL_DRY_ (%)	PL_WET_ (%)	BD (%)	Performance Score
PA1	0.99	0.97	0.76	0.79	0.68	0.39
PA2	0.97	0.95	0.92	0.85	0.37	0.27
PA3	0.96	0.94	0.85	1.00	0.28	0.22
PA4	0.99	0.98	0.80	0.67	0.68	0.35
PA5	0.97	0.96	0.89	0.85	0.57	0.40
PA6	0.97	0.95	1.00	0.94	0.35	0.30
PA7	1.00	1.00	0.71	0.69	0.61	0.30
PA8	0.98	0.97	0.75	0.77	0.68	0.38
PA9	0.95	0.93	0.92	0.98	0.43	0.34
PA10	0.99	0.98	0.76	0.87	1.00	0.64
PA11	0.97	0.95	0.81	0.91	0.46	0.31
PA12	0.98	0.97	0.78	0.89	0.32	0.21
PA13	0.98	0.97	0.77	0.84	1.00	0.61
PA14	0.98	0.96	0.83	0.92	1.00	0.72
PA15	0.97	0.95	0.87	0.94	0.35	0.26
PA16	0.98	0.97	0.79	0.82	1.00	0.61
PA17	0.98	0.96	0.82	0.93	1.00	0.72
PA18	0.97	0.96	0.91	0.99	0.46	0.38

**Table 13:** Analysis of variance results for JPS.

Source	DF	Adj SS	Adj MS	F-Value	P-Value
Model	6	0.18	0.03	5.79	0.006
Linear	3	0.11	0.04	6.94	0.007
FC (%)	1	0.01	0.01	2.23	0.164
BC (%)	1	0.01	0.01	2.22	0.165
FT	1	0.09	0.09	16.39	0.002
Two-Way Interaction	3	0.08	0.03	4.90	0.021
FC (%)*BC (%)	1	0.01	0.01	2.12	0.174
FC (%)*FT	1	0.01	0.01	2.80	0.123
BC (%)*FT	1	0.05	0.05	9.79	0.010
Error	11	0.06	0.01		
Total	17	0.24			
